# Optimal Duration of Drought Stress Near Harvest for Promoting Bioactive Compounds and Antioxidant Capacity in Kale with or without UV-B Radiation in Plant Factories

**DOI:** 10.3390/plants9030295

**Published:** 2020-03-01

**Authors:** Hyo In Yoon, Wenjuan Zhang, Jung Eek Son

**Affiliations:** Department of Plant Science and Research Institute of Agriculture and Life Sciences, Seoul National University, Seoul 08826, Korea; yoonhi@snu.ac.kr (H.I.Y.); coconutpalm02@126.com (W.Z.)

**Keywords:** antioxidant capacity, drought stress, flavonoid, kale, phenolic compound, UV radiation

## Abstract

Among abiotic stresses, both drought and UV-B radiation effectively trigger the accumulation of secondary metabolites, and can be widely applied in plant factories. The objectives of this study were to investigate antioxidant accumulation under drought stress alone, or in combination with UV-B radiation near harvest, and to determine an optimal treatment time for maximum antioxidant production. Kale (*Brassica oleracea* L. var. *acephala*) plants were grown in a plant factory and harvested at 42 days after transplanting. The single and combination treatments lasted for 7 to 1 days and 4 to 2 days before harvest, respectively. The results of both F_v_/F_m_ (maximal photochemical efficiency in photosystem II) and leaf water potential could ensure the function of photosynthesis and maintain normal leaf moisture in single drought treatments of less than 4 days. The total phenolic and flavonoid contents and antioxidant activities were significantly increased in both single and combination treatments for 3 to 4 days, compared to other treatments. The supplementary UV-B treatments showed no extra formation of antioxidants compared to the single drought treatments. As a result, drought for 3 days before harvest could achieve the highest potential value of kale as a source of natural antioxidants.

## 1. Introduction

Kale (*Brassica oleracea* L. var. *acephala*) is an excellent source of dietary antioxidants, and is commonly cultivated around the world. The consumption of kale is increasing as a source of functional foods and nutraceuticals, of which the protective health effects are attributed to its radical scavenging and metal-chelating abilities [1]. The production of phytochemical compounds is often low (less than 1% dry weight), and depends greatly on the physiological and developmental stage of the plant [2,3,4]. The qualitative and quantitative compositions of phytochemical compounds vary depending on multiple factors. For example, maturity, climate and farming practices have been shown to affect the carotenoid contents of kale, such as lutein and zeaxanthin [5].

Abiotic stressors, such as high and low temperatures, drought, alkalinity, salinity and ultraviolet (UV) radiation, have been shown to enhance the potential to overproduce useful phytochemicals, and thereby enhance the accumulation of antioxidants [6]. Drought is one of the effective abiotic stressors that can enhance a number of phytochemical compounds, including α-tocopherol, β-carotene and flavonoids, in a wide range of plant species [7,8]. 

UV-B radiation is another stressor that has been linked to a broad range of metabolites, including phenolic, terpenoid and alkaloid compounds [9,10]. Combined effects of both drought and UV-B radiation can modify the response patterns, but the effects depend upon the species or cultivars, the intensity of stressors and the duration of exposure [11]. Stressors can act either synergistically, or to some extent antagonistically, in terms of inducing plant protective mechanisms. In previous studies, the interaction of drought and UV-B radiation increased salicylic acid accumulation in barley leaves, and proline content and PAL activity in lettuce, but decreased quercetin in lettuce compared to single treatment [12,13]. 

Controlled environments, such as plant factories, can be used to determine the levels of multiple factors affecting phytochemical compounds. However, most studies of abiotic stresses have been applied to plant seedlings, young leaves or organs [14,15,16]. For functional food production, the effect of the stresses on whole, mature plants and the quantitative analysis of these stressors to determine the optimal treatments, are required. Optimal strategies for drought treatment with or without UV-B radiation near harvest, which can contribute to the maximum production of antioxidants in plants, have not been established. The objectives of this study were to investigate the effects of treatment time under drought stress alone, or in combination with UV-B radiation near harvest on the growth, photosynthetic trait and antioxidant accumulation of kale in a plant factory, and to determine an optimal treatment time for maximum antioxidant production. We investigated the growth, chlorophyll fluorescence, leaf water potential, phenolic and flavonoid compounds accumulations and antioxidant activity, as the physiological and biochemical responses of kale.

## 2. Materials and Methods 

### 2.1. Plant Materials and Growth Conditions

Kale seeds (*Brassica oleracea* L. var. *acephala*, ‘Manchoo collard,’ Asia Seed Company, Seoul, Korea) were sown on sponge cube and germinated in deep flow systems at a photosynthetic photon flux density (PPFD) of 150 μmol m^−2^ s^−1^ irradiated by fluorescent lamps. At one week after germination, the nutrient solution for *Brassica* [17] was applied with an electrical conductivity (EC) of 0.6 dS m^−1^. After the fourth normal leaves appeared, seedlings of uniform size were transplanted into a plant factory module with a deep flow technique system (with an air temperature of 20 °C; relative humidity of 70% ± 5%; CO_2_ concentration of 500 ± 5 μmol·mol^−1^; PPFD of 350 μmol m^−2^ s^−1^ irradiated by light-emitting diodes (LEDs) of red:blue:white = 8:1:1; light period of 16 h). The nutrient solutions were supplied with an EC of 1.2 dS m^−1^. Three plants per treatment were harvested at 42 days after transplanting (DAT), which is the optimal harvest time for maximizing the annual production of bioactive compounds in this cultivar [4]. After the whole leaves were lyophilized with a freeze dryer (FD8512, Ilshin Biobase Co., Yangju, Korea) at −80 °C under a vacuum of 0.007 mm Hg for 120–168 h, and the shoot dry weight (DW) was measured with three replicates.

### 2.2. Drought and UV-B Treatments

**Figure 1:**
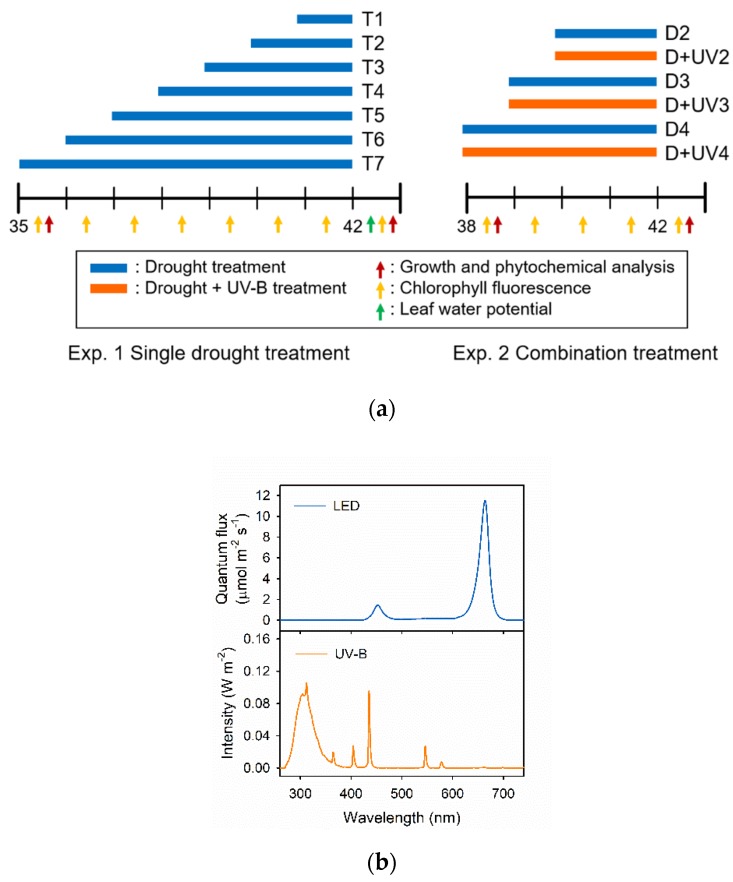
(**a**) Experimental design; (**b**) light spectra of the light emitting diode (LED) and UV-B tube. In experiments 1 and 2, the plants were harvested 42 days after transplanting (DAT). Drought treatments were imposed by releasing all of the nutrient solutions from the root system (blue bar). Combination treatment groups were subjected to drought with UV-B exposure (orange bar). The arrows indicate the type and date of each measurement.

In Exp. 1, the drought stress was imposed by removing all of the nutrient solution from the root system in the growing modules, lasting for 7, 6, 5, 4, 3, 2 and 1 days before harvest (T7, T6, T5, T4, T3, T2 and T1, respectively). In Exp. 2, the drought stress alone and in combination with UV-B radiation were applied for 4, 3 and 2 days before harvest (D4, D3, D2, D+UV4, D+UV3 and D+UV2, respectively). The combined treatments were exposed to a UV-B lamp (Sankyo Ultraviolet Co. Ltd., Kanagawa, Japan) from 10:00 to 14:00. The UV-B dose was 4.2 W m^−2^ (60.5 kJ m^−2^ d^−1^). Light spectra were measured using a spectroradiometer (Blue-Wave spectrometer, StellarNet Inc., Tampa, FL, USA) with a range of 280 to 800 nm. For the control group, no stress was applied. The experimental setup is described in Figure 1. 

### 2.3. Leaf Water Potential

Leaf water potential (Ψ_l_) was measured at 42 DAT using a dewpoint potentiometer (WP4, Decagon Devices, Inc., Pullman, WA, USA), according to the method recommended by the instrument manufacturer. Before all measurements, the leaf surface was wetted with a drop of distilled water, abraded with ultrafine sandpaper (50 × 20 mm, 600-grit) and then blot dried with lint-free tissues (Kimwipes, Kimtech Science, Roswell, GA, USA), to remove any excess moisture. The leaf disks were collected daily with a circle of 4 cm diameter, and were measured with three replicates. Equilibration for each measurement reached was typically within 30 min, and the results of Ψ_l_ were expressed in megaPascals (Mpa).

### 2.4. Chlorophyll Fluorescence

Chlorophyll fluorescence was measured under laboratory conditions (ambient CO_2_ conditions) with a chlorophyll fluorescence meter (Handy PEA, Hansatech Instruments, Kings Lynn, UK). The measurements were taken on a fully expanded leaf from the middle of the canopy, with three replicates in each treatment. Adaptation in darkness for 30 min was preceded using a leaf clip (HPEA/LC, Hansatech Instruments, Kings Lynn, UK). The measurements were performed using a saturating pulse of 1500 μmol m^−2^ s^−1^ (a pulse duration of 1 s and a fixed gain of 1.5×) to determine the minimal fluorescence (F_0_) and maximal fluorescence (F_m_). The maximal photochemistry efficiency of photosystem II (F_v_/F_m_) was calculated as (F_m_ − F_0_)/F_m_.

### 2.5. Sample Extraction

After harvest, three plants per treatment were lyophilized with the freeze dryer at −80 °C under a vacuum of 0.007 mm Hg for 120–168 h. The freeze-dried leaves were ground to a fine powder with the aid of a mortar and pestle. The samples were assumed to be uniform. Each powdered sample (approximately 100 mg) was mixed with 1 mL of 70% (*v*/*v*) aqueous methanol. The samples were incubated for 48 h in darkness at room temperature (for phenolic and antioxidant activity analysis), or for 12 h in darkness at 4 °C (for flavonoid analysis). Before each analysis, the samples were centrifuged at 1.0 × 10^4^ *g* for 10 min.

### 2.6. Total Phenolic Determination

Total phenolic contents (TPCs) were determined according to the Folin–Ciocalteu colorimetric method [18]. The supernatant of sample (50 μL) was mixed with 750 μL of 10% Folin–Ciocalteu reagent and 135 μL of distilled water. After vortex mixing, 600 μL of 700 mM Na_2_CO_3_ was added, and the sample was incubated for 2 h at room temperature. The aqueous methanol (50 μL) was used as a blank instead of the sample. The absorbance of the sample at 765 nm was measured with a spectrophotometer (Photolab 6100vis, WTW, Weilheim, Germany). The TPCs were expressed as mg of gallic acid equivalents per g of DW (mg GAE g^−1^ DW).

### 2.7. Flavonoid Determination

Total flavonoid compounds (TFCs) were measured using a colorimetric method [19,20]. The supernatant (150 μL) was collected and added to 135 μL of distilled water and 45 μL of 5% NaNO_2_. After 5 min at room temperature, the sample was mixed with 90 μL of 10% AlCl_3_ and incubated for 6 min at room temperature. Then, 300 μL of 1 M NaOH and 165 μL of distilled water were added, and the sample was incubated for 5 min. The absorbance of the sample at 510 nm was measured with the spectrophotometer. The TFCs were expressed as mg of catechin acid equivalents per g of DW (mg CE g^−1^ DW).

### 2.8. Antioxidant Activity Analysis

In vitro antioxidant capacity was measured using the 2,2-diphenyl-1-picrylhydrazyl (DPPH) free radical scavenging assay [21,22]. The DPPH stock solution was prepared by dissolving 24 mg of DPPH with 100 mL of 95% methanol. The supernatant (150 μL) was collected and mixed with 1.35 mL of DPPH solution, and incubated for 30 min at room temperature. The absorbance of the sample at 517 nm was measured using methanol as a blank with the spectrophotometer. The results were expressed as mg of ascorbic acid equivalent antioxidant capacity per gram of DW (mg AEAC g^−1^ DW).

### 2.9. Statistical Analysis

The experiment was performed using a completely randomized block design. All measurements were replicated three times. The results were compared via a one-way analysis of variance (ANOVA), followed by Tukey’s multiple comparison test at *p* < 0.05 using Sigmaplot 12.5 (Systat Software Inc., San Jose, CA, USA).

## 3. Results and Discussion

### 3.1. Leaf Water Potential and Chlorophyll Fluorescence in Single Drought Stresses

**Figure 2:**
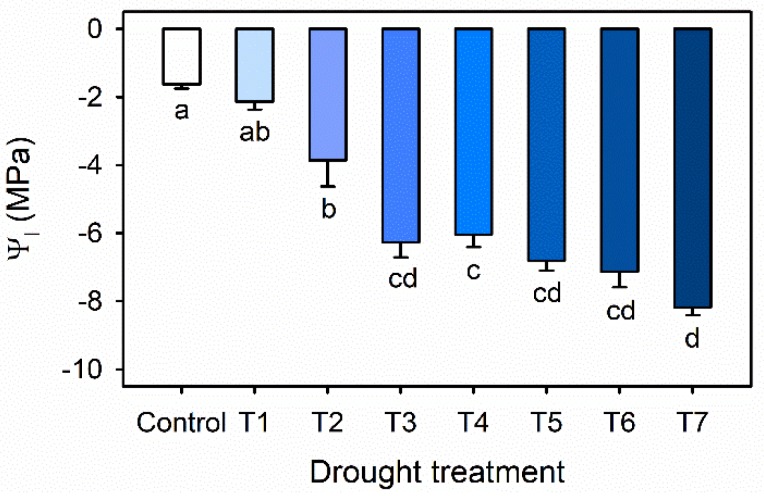
Leaf water potential (Ψ_l_) of kale under the control and drought stress treatments started 7, 6, 5, 4, 3 or 1 days before harvest (T7, T6, T5, T4, T3 and T1). The error bars represent one standard deviation (SE); *n* = 3. The different letters indicate significant differences according to the Tukey test (*p* < 0.05).

Leaf water potential (Ψ_l_) was gradually decreased with increasing drought stress duration in Exp. 1 (Figure 2). Ψ_l_ was significantly decreased from −3.86 MPa in T2 to −6.27 MPa in T3 and until −8.19 MPa in T7. The results were in agreement with previous reports, which showed that leaf water potentials of water-stressed blueberry were gradually decreased with the level of water stress [23,24].

In this result, the lowest Ψ_l_ of −8.19 MPa was similar to the lethal water potential of pigeon pea (*Cajanus cajan*), which ranged from −6.8 to −8.2 Mpa, and indicates dehydration resistance [25]. In this study, severe drought stress resulted in turgor loss, and the plants appeared to be wilty. 

**Figure 3:**
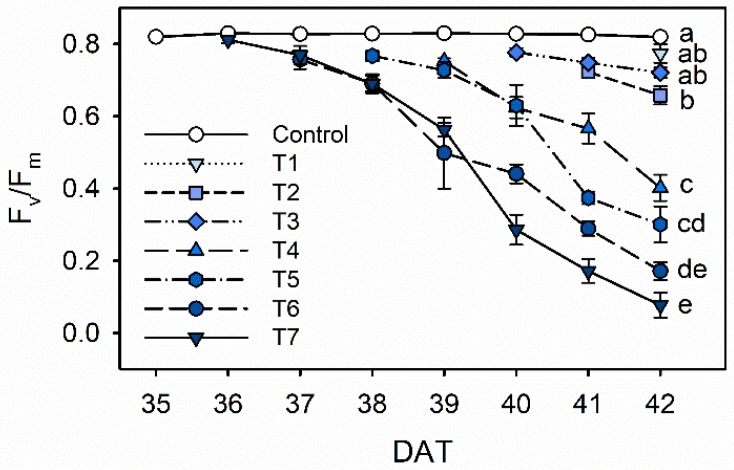
The maximal photochemistry efficiency of PSII (F_v_/F_m_) of kale under the control and drought stress treatments started 7, 6, 5, 4, 3 or 1 days before harvest (T7, T6, T5, T4, T3 and T1, respectively). The error bars represent 1 SE; *n* = 3. The different letters indicate significant difference at harvest according to the Tukey test (*p* < 0.05).

Chlorophyll fluorescence (F_v_/F_m_) steadily decreased with increasing drought stress during 35 to 42 DAT in Exp. 1 (Figure 3). The F_v_/F_m_ values of 0.77–0.72 in T1 and T3 did not differ significantly compared to the control value of 0.82. The F_v_/F_m_ significantly decreased from 0.40 in T4 to 0.08 in T7. In the T7 treatment, the F_v_/F_m_ showed a drastic decline during 39 and 40 DAT (0.56 and 0.29, respectively), which are 4 and 5 days after the drought stress started. Similarly, Fang et al. showed that *Caragana korshinskii* water stressed by withholding water significantly decreased photosynthesis and electron transport in photosystem II (PSII) with longer water stress [26]. A decrease in F_v_/F_m_ under drought stress has been shown in many plants, such as wheat, lettuce, oil palm and grapevine [15,27,28,29]. These changes in quantum yield resulted from downregulation of PSII activity, due to an imbalance between light capture and its utilization under drought stress [30].

Along with the decrease observed in leaf water potential (Ψ_l_), the decreases in the maximal photochemistry efficiency of PSII (F_v_/F_m_) indicated that kale leaves experienced a certain degree of stress during the period (Figure 2 and Figure 3). Under water stress, Ψ_l_ is a more sensitive indicator than chlorophyll fluorescence parameters, including F_v_/F_m_ [28]. In this study, drought stress over 4 days dramatically reduced chlorophyll fluorescence. According to both results, drought stress treatments were shortened to 1 to 4 days before harvest to determine the optimal treatment duration.

### 3.2. Bioactive Compounds, Antioxidant Activity, and Plant Growth in Single Drought Stresses

**Figure 4:**
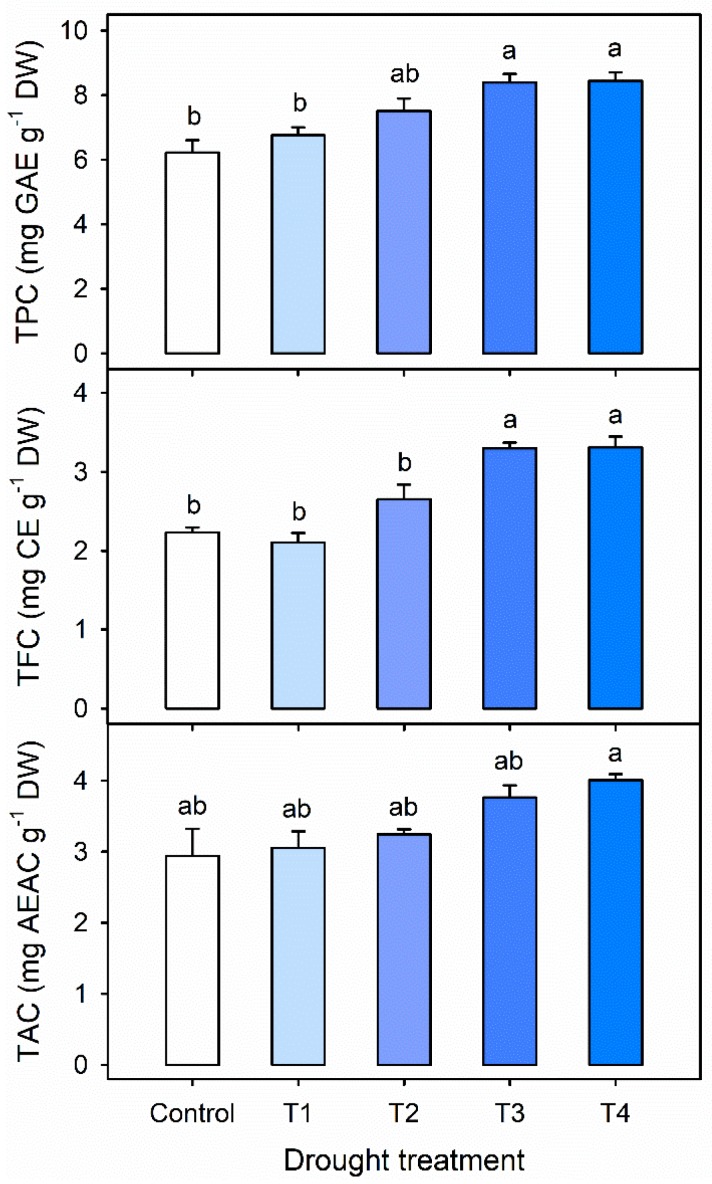
Total phenolic content (TPC), total flavonoid content (TFC), and total antioxidant capacity (TAC) per dry weight in kale leaves at 42 days after transplanting (DAT) under the control and single drought stress treatments started 4, 3, 2 or 1 days before harvest (T4, T3, T2 and T1, respectively). The error bars represent 1 SE; *n* = 3. The different letters indicate significant difference according to the Tukey test (*p* < 0.05).

The concentrations of TPC, TFC and TAC were higher with longer drought stress in Exp. 1 (Figure 4). The concentrations of TPC and TFC were significantly increased by 35% and 48%, respectively, in both T3 and T4 compared to the control. The total antioxidant capacity (TAC) in T4 was significantly higher than that in the control. The results were in agreement with a previous report by Oh et al., which showed that the drought stress induced by withholding water for 4 days improved the TPC and TAC in lettuce [15]. Furthermore, numerous studies have shown that drought stress enhances one or both enzymatic and nonenzymatic antioxidants in a wide range of plants [31,32,33,34,35]. Moderate drought stress can improve plant drought tolerance, and enhance the antioxidant system [30,36]. In this study, the response of kale to drought stress was highly dependent upon the stress period before harvest. The results suggest that the drought stress lasting 3 to 4 days can improve the accumulation of antioxidants in kale.

The shoot DWs were not significantly different with drought stresses for 1 to 3 days in Exp. 1 (Table 1). Only the DW in T4 was significantly decreased by 8.0% compared to that of the control. The accumulations of TPC and TFC were significantly increased 25% and 37%, respectively, in both T3 and T4 compared to those in the control. The TAC per plant was not significantly increased with drought stress. The changes in antioxidant capacity under drought stress are different among plant species. The DPPH free radical scavenging activity was not changed in *Limonium* species, despite water deficit stress for 1 month [34]. However, the same activity was significantly increased in St. John’s wort plants after water stress for 12 days [8]. In the present study, the accumulation per plant was obtained by multiplying the dry weight of whole leaves with the concentration of antioxidants. Considering both the concentration of nutrients and dry matter yield, the dilution effect in plant nutrients can be taken into account [37]. Additionally, the accumulation per plant can be used to estimate the annual production of bioactive compounds and total antioxidant activities [4]. The results showed that the optimal drought duration in kale was 3 days before harvest for maximizing TPC and TFC without growth loss.

### 3.3. Chlorophyll Fluorescence in Combined Drought and UV-B Radiation Stresses

**Figure 5:**
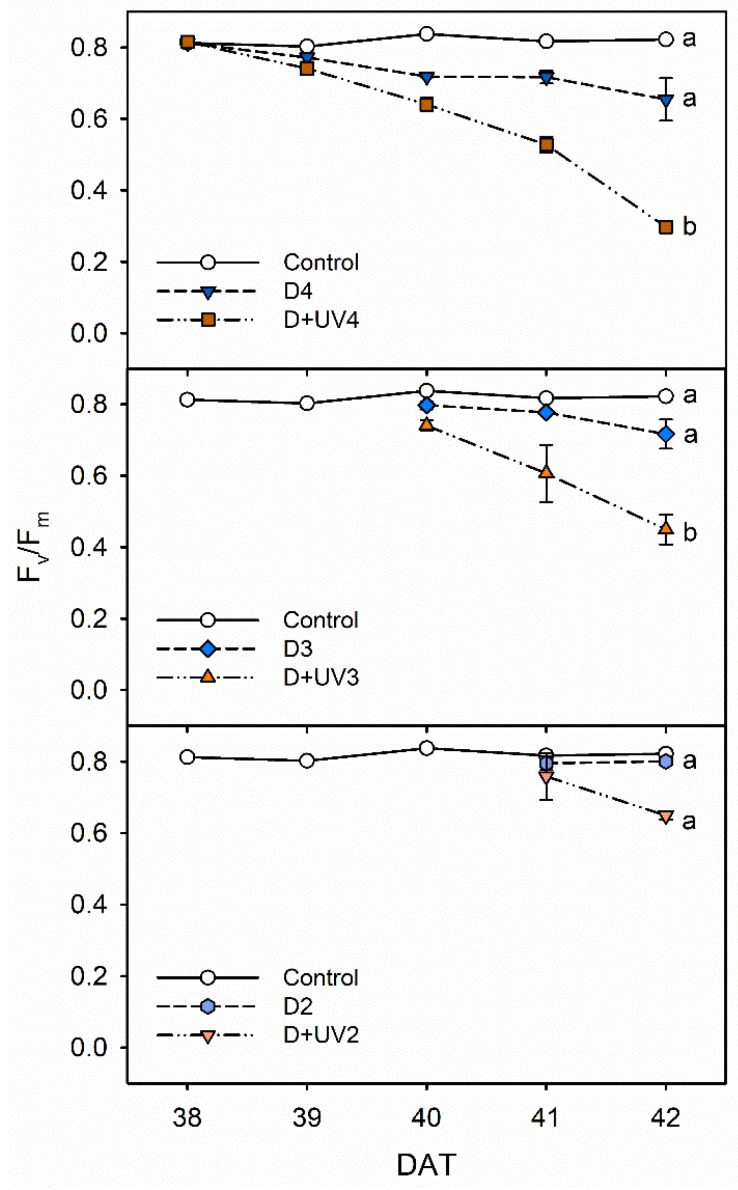
The maximal photochemistry efficiency of PSII (F_v_/F_m_) of kale under control and drought stress alone and in combination with UV-B radiation started at 4, 3 and 2 days before harvest (D4, D3, D2, D+UV4, D+UV3 and D+UV2, respectively). The error bars represent 1 SE; *n* = 3. The different letters indicate significant difference at harvest according to the Tukey test (*p* < 0.05).

The F_v_/F_m_ values in combined drought and UV-B radiation stresses for 3 to 4 days were significantly lower than those in single drought stress and the control in Exp. 2 (Figure 5). The combined stress for 2 days did not significantly affect chlorophyll fluorescence. The results suggest that the photosynthetic efficiency in PSII is more sensitive to UV-B radiation than to drought stress in kales. The results were in agreement with a previous report by Martínez-Lüscher et al., which showed that the F_v_/F_m_ under water deficit and UV-B exposure was lower than that under water deficit alone in grapevine plants [29]. In addition, PSII has greater resilience to water deficit compared to PSI [38,39]. However, the location on the plant upon which the stressor acts may also be relevant. The top of the plants was exposed to UV-B radiation. Water deficit affected the whole leaves from the bottom to the top, as indicated by the F_v_/F_m_ distribution [40].

### 3.4. Bioactive Compounds, Antioxidant Activity, and Plant Growth in Combined Stresses

**Figure 6:**
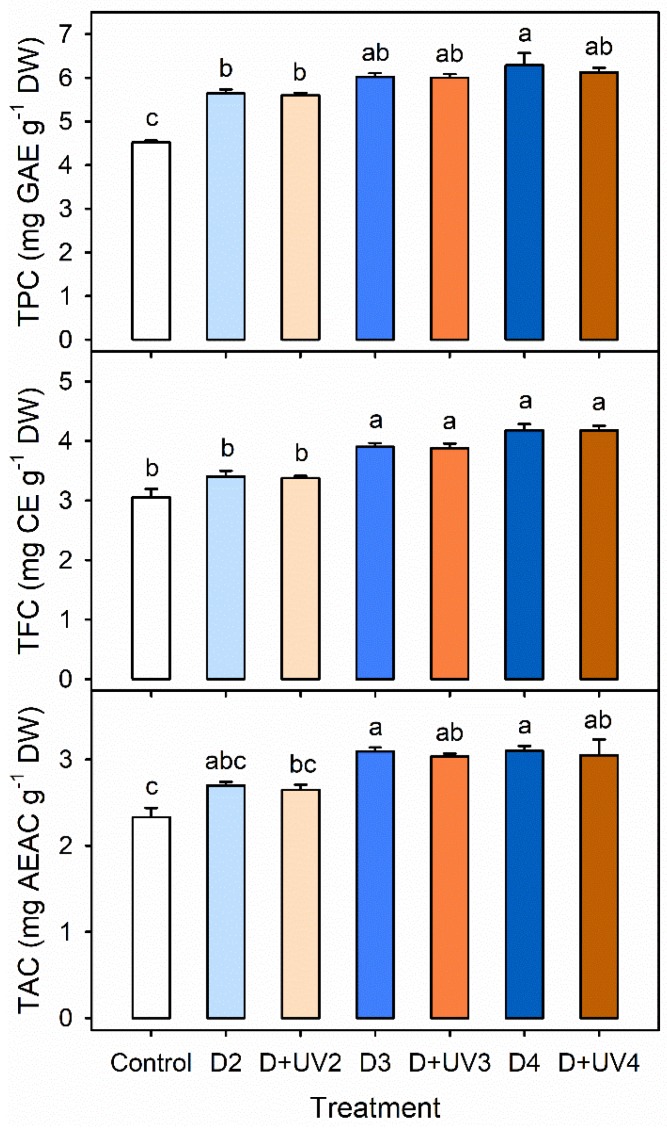
Total phenolic content (TPC), total flavonoid content (TFC), and total antioxidant capacity (TAC) per dry weight in kale leaves at 42 DAT under control and drought stress alone and in combination with UV-B radiation started at 4, 3 and 2 days before harvest (D4, D3, D2, D+UV4, D+UV3 and D+UV2, respectively). The error bars represent 1 SE; *n* = 3. The different letters indicate significant difference according to the Tukey test (*P* < 0.05).

The concentrations of TPC, TFC and TAC were higher with longer drought stress. with or without UV-B radiation in Exp. 2 (Figure 6). The concentrations of TPC and TFC were significantly increased in D3, D4, D+UV3 and D+UV4, compared to those in the control. The concentration of TPC in D4 was highest among all treatments, and significantly higher than those in D2 and D+UV2. The antioxidant capacities in D3, D4, D+UV3 and D+UV4 were significantly increased, compared to that in the control. At all concentrations, the combined stress treatments were not significantly different from the single drought stress with the same duration.

The shoot DWs were not significantly different with longer drought stress with or without UV-B radiation, except for D+UV4 in Exp. 2 (Table 2). Only the DW in D+UV4 was significantly decreased by 8.8%, compared to that of the control. Similarly, Lee et al. showed that the fresh weight of lettuce plants and F_v_/F_m_ decreased with longer UV-B exposure [41]. UV-B radiation may enhance resilience to drought stress, and vice versa [11]. In treatments combining drought and UV-B radiation, the dry matter values were higher than those under UV-B radiation alone in plants, such as lettuce, pea and wheat [13,42].

The accumulations of TPC, TFC and TAC were higher with longer drought stress with or without UV-B radiation (Table 2). The TPC accumulations were significantly increased under the single and combined stresses compared to that in the control. The TFC and TAC accumulations were also significantly increased under the stresses, except for D2 and D+UV2. The TPC and TFC accumulations in D4 were the highest, and increased by 30.9% and 28.7%, respectively, compared to those in the control. As the result, the optimal drought and UV-B radiation duration in kale was 3 days before harvest for maximizing TPC and TFC without growth loss. UV-B treatments imposed additional energy costs, and therefore, the single drought treatment for 3 days was more effective than the combination treatment.

In this result, supplemental UV-B exposure to kale did not produce any extra accumulation of TPC, TFC and TAC compared with the drought alone, which was consistent with the response in lettuce plants [13]. In contrast, Bandurska et al. showed that the accumulations of phenolic acids, proline and UV-B absorbing compound in response to a combination of water deficit and UV-B radiation were higher than those in the single drought stress in barley leaves [43]. Both stresses can act synergistically to induce protective mechanisms [44]. Proline production is known to occur under both water deficit and UV-B radiation, contributing to stabilizing subcellular structures, scavenging free radicals and buffering cellular redox potentials [45]. As abiotic stresses, drought and UV-B radiation enhance many secondary metabolites; however, excessive stresses cause adverse effects on plant growth and productivity [6,30]. The plant response to a stress highly depends upon the species, cultivar, plant organ, developmental stage, as well as eco-physiological interactions [43,46]. In addition, the signaling pathway in the plant response to drought and UV-B radiation differently affects the cellular defense mechanism [11]. Therefore, the antioxidant accumulation under the interaction of drought and UV-B radiation is difficult to predict.

## 4. Conclusions

This study compared the effects of drought stress alone and in combination with UV-B radiation near harvest on growth, chlorophyll fluorescence, and antioxidant accumulations, and determined the optimal treatment time in kale. Drought beginning less than 4 days before harvest ensured the function of photosynthetic efficiency in PSII and maintained normal leaf water potential. Drought stress lasting 3 to 4 days can improve the concentrations of antioxidants in kale. Considering the loss of growth, the optimal drought duration in kale was 3 days before harvest. The combination of drought and UV-B for 3 to 4 days significantly reduced the F_v_/F_m_ at harvest. Combined stresses showed no additional production of antioxidants compared to those obtained under single drought stress in this study. Considering energy costs, drought stress lasting 3 days before harvest could achieve the highest potential value of kale as a source of natural antioxidants.

## Figures and Tables

**Table 1:** Shoot dry weight (DW), total phenolic content (TPC), total flavonoid content (TFC) and total antioxidant capacity (TAC) per plant in kale leaves at 42 DAT under control and drought stress treatments started 4, 3, 2, or 1 days before harvesting (T4, T3, T2, and T1, respectively).

Treatment	Shoot DW (g)	TPC Per Plant (mg GAE plant^−1^)	TFC Per Plant (mg CE Plant^−1^)	TAC Per Plant (mg AEAC Plant^−1^)
Control	10.55 a ^1^	65.61 b	23.55 b	22.35 a
T1	10.34 ab	69.83 ab	21.77 b	31.03 a
T2	10.10 ab	75.88 ab	26.80 ab	31.54 a
T3	9.82 ab	82.35 a	32.37 a	32.77 a
T4	9.71 b	81.89 a	32.15 a	36.94 a

^1^ Mean (*n* = 3); different letters (a, b) indicate significant differences according to the Tukey test (*p* < 0.05).

**Table 2:** Shoot dry weight (DW), total phenolic content (TPC), total flavonoid content (TFC) and total antioxidant capacity (TAC) per plant in kale leaves at 38 DAT (Before) and 42 DAT under control and drought stress alone and in combination with UV-B radiation started at 4, 3 and 2 days before harvest (D4, D3, D2, D+UV4, D+UV3 and D+UV2, respectively).

Treatment	Shoot DW (g)	TPC Per Plant (mg GAE plant^−1^)	TFC Per Plant (mg CE Plant^−1^)	TAC Per Plant (mg AEAC Plant^−1^)
Control	11.49 a ^1^	51.98 c	35.11 c	26.78 c
D2	11.26 a	63.57 ab	38.31 bc	30.40 abc
D3	11.09 ab	66.80 ab	43.29 a	34.33 a
D4	10.81 ab	68.03 a	45.19 a	33.51 ab
D+UV2	10.94 ab	61.25 b	36.96 c	28.96 bc
D+UV3	10.80 ab	64.82 ab	41.87 ab	32.80 ab
D+UV4	10.48 b	64.16 ab	43.77 a	31.95 ab

^1^ Mean (*n* = 3); different letters (a–c) indicate significant differences according to the Tukey test (*P* < 0.05).
